# Description and Application of a Mathematical Method for the Analysis of Harmony

**DOI:** 10.1155/2015/831396

**Published:** 2015-06-16

**Authors:** Qiting Zuo, Runfang Jin, Junxia Ma, Guotao Cui

**Affiliations:** College of Water Conservancy and Environment, Zhengzhou University, No. 100, Science Road, Zhengzhou 450001, China

## Abstract

Harmony issues are widespread in human society and nature. To analyze these issues, harmony theory has been proposed as the main theoretical approach for the study of interpersonal relationships and relationships between humans and nature. Therefore, it is of great importance to study harmony theory. After briefly introducing the basic concepts of harmony theory, this paper expounds the five elements that are essential for the quantitative description of harmony issues in water resources management: harmony participant, harmony objective, harmony regulation, harmony factor, and harmony action. A basic mathematical equation for the harmony degree, that is, a quantitative expression of harmony issues, is introduced in the paper: HD = *ai* − *bj*, where *a* is the uniform degree, *b* is the difference degree, *i* is the harmony coefficient, and *j* is the disharmony coefficient. This paper also discusses harmony assessment and harmony regulation and introduces some application examples.

## 1. Introduction

With the exception of “goodwill competition,” living in harmony (in terms of the relationships between people) is recommended, and the resulting community of people living in harmony is often called a “harmony society,” “harmony community,” “harmony city,” “harmony home,” and “harmony team.” From the point of view of relationships between humans and nature, it is impossible for human beings to dominate nature because people would be forced to live in harmony with nature as a result of a nature counterattack. Therefore, there is no doubt that human beings and nature should be harmonious.

When the word “harmony” is mentioned, it is often associated with the word “games.” Game theory is concerned with the behavior of absolutely rational decision makers with unlimited capabilities for reasoning and memorization [1]. Games are defined mathematical objects that consist of a set of players, a set of strategies (i.e., options or moves) that are available to the players, and a specification of the payoff that each player receives for each combination of strategies (i.e., possible outcomes of the game) [2]. Game theory has been used in a variety of fields, and it includes many contents in each field. For example, in water resources research, it reflects in lots of ways, including allocation of water resources [3, 4], water rights [5], water resources development [6], optimal allocation of water resources [7–9], problems of water environment [10], water resources management [11–13], and water conflicts [14, 15]. Game theory is used to represent the “struggle or competition” phenomenon and can be frequently encountered in practice, such as bargaining, offensive and defensive battles, horse racing, and auctions. However, it is insufficient just considering the games. Games can only be used to represent a struggle or competitive phenomenon. In contrast, it is necessary to build a harmony balance in many situations, and game theory cannot be applied for common harmony issues. In addition, there are some extraordinarily difficult problems, such as the “tragedy of the commons” [16, 17], which cannot be solved by game theory alone.

In game theory, “the tragedy of the commons” has been mentioned in the literature through various expressions, but the meaning is basically the same. The “tragedy of the commons” roughly means as follows: if there is a set piece of grassland that is shared by two homes for sheep grazing, the total number of sheep is limited due to the limited grass. From the point of view of the individual, a home that raises more sheep will have a better profit. To maximize his/her profits, each individual attempts to increase his/her number of sheep, which results in an increasingly high number of total sheep and thus an increasingly excessive use of the grass. This excess leads to grassland degradation and even destruction, that is, the “tragedy of the commons.” Therefore, in some cases, it is insufficient to only consider game theory; there is a need to consider harmony issues in these cases. As a result, harmony theory should also be established.

This paper has three objectives: (1) to introduce the concepts of harmony theory and the five essential elements of harmony theory in water resources management based on the above analysis and previous studies [18]; (2) to discuss the mathematical description of harmony theory by proposing a function for the harmony degree, introducing a mathematical approach for the assessment of harmony, and developing a method for harmony regulation; (3) to illustrate the mathematical description of harmony by a series of typical examples.

## 2. Concepts

Although the word “harmony” is widely used, a unifying concept has not yet been defined. Harmony in this paper is defined as follows: harmony is the action taken to achieve “coordination, accordance, balance, integrity, and adaptation.” Because people rely on nature to survive, it is necessary for human society to live in harmony with nature.

The theory and methodology of studies on harmony behavior are termed harmony theory, which is further defined as follows: harmony theory is a method through which various participants work together to achieve harmony. Harmony theory, which is of broad application prospect, is a significant theory that reveals the harmonious relationships in nature and is also a concrete manifestation of dialectical materialism on the assertion of “the coordinated development between humans and nature.” Firstly, it should be recognized that “harmony is an important concept in addressing interpersonal relationships and relationships between humans and nature, and it is also a major guarantee and a concrete manifestation to build a harmony society, harmony community, harmony team, and harmony nature.” Secondly, it is important to gradually establish the concept of harmony and adhere to the ideological philosophy of harmony. In addition, humans should take the initiative to coordinate the marvelous relationships between people, which is the basis for the coordination of relationships between humans and nature. Furthermore, it is a new theory, and it can provide an appropriate pathway for water resources management in China [19]. The main arguments of harmony theory are the following.

1. Harmony theory advocates the philosophy that “harmony is the most precious” to address a variety of relationships, and harmony ideology is the cornerstone of harmony theory.
2. Harmony theory advocates a rational understanding of various contradictions and conflicts existing in various types of relationships, allowing the existence of differences and promoting a harmonious attitude to address various factors of disharmony and problems. Instead of ignoring the disharmony factors, it is necessary to consider all of the harmony factors and disharmony factors.
3. Harmony theory advocates the concept of harmony between humans and nature and has very pronounced views on the coordinated development of these relationships. It asserts that human beings should take the initiative to coordinate the marvelous relationships among people. There is a possibility to achieve the coordination of the relationships between humans and nature based on this theory.
4. Harmony theory adheres to the system perspective by promoting system-wide theoretical methods to study the issues of harmonious relationship.

## 3. Five Factors of Harmony Theory

To obtain a reasonable expression of harmony and a quantitative description of the harmony degree, the following five elements, which are the “five essential factors of harmony theory,” need to be defined [18].

*(1) Harmony Participant*. The term “harmony participant” refers to the parties (generally two or more) involved in the harmony relationship, which are known as “the harmony party.” The collection of harmony participants can be represented as *H* = {*H*_1_, *H*_2_,…, *H*_*n*_}, where *n* is the number of participants in the harmony party, which is also named “*n*-participant harmony.” For a certain harmony party, this variable can be expressed as *H*_*k*_  (*k* = 1,2,…, *n*). For instance, the participants of a harmonious couple are the two spouses, and the harmony participants of a family are all of the family members.

*(2) Harmony Objective*. This term refers to the target that the harmony participants have to achieve a state of harmony. If not, it is impossible to arrive at a state of harmony. In addition, attaining this goal might only lead to a partial state of harmony. For example, if there are *n* families sharing a piece of meadow for sheep, it is imperative to ensure that the total number of sheep does not exceed a certain amount (i.e., stocking rate) to avoid grass damage; the certain amount is thus the harmony target of the *n* households that share a piece of grassland.

*(3) Harmony Regulation*. This term refers to all of the rules or constraints established by the participants for the purpose of achieving the harmony goals. For example, in order to ensure rationality, a harmony regulation for the abovementioned *n* households sharing a piece of grassland could be that the amount of the increase in sheep for each household should be proportional to their population. Thus, according to the conditions of these harmony rules, it is appropriate to study harmony problems.

*(4) Harmony Factor*. This term refers to the factor that should be considered by harmony participants to achieve overall harmony. Its collection is represented as *F* = {*F*_1_, *F*_2_,…, *F*_*m*_}, where the *p*th harmony factor is *F*_*p*_ and the total number of factors is *m*. When *m* = 1, it indicates single-factor harmony, and the harmony factor can be directly expressed as *F*. If *m* ≥ 2, the harmony relationship is called multiple-factor harmony.

*(5) Harmony Action*. The term “harmony action” refers to the general name of the concrete behavior of the harmony participants for the harmony factors. For example, if *n* households jointly own a field of grass, the specific action is the quantity of sheep that are raised on that land. The collection of harmony actions taken by the participants in the *n*-participant harmony and the *m* harmony factors can be expressed as a matrix:

(1) A11,A12,…,A1nA21,A22,…,A2nkk⋮Am1,Am2,…,Amn.

A single-factor harmony action is represented as *A* = {*A*_1_, *A*_2_,…, *A*_*n*_}.

## 4. Calculation of the Harmony Degree

The harmony degree is used for the quantitative expression of the harmony degree [18]. In this section, the harmony degree equation of a given factor (*F*_*p*_) will be introduced, (i.e, Zuo-harmony degree equation). Then, the calculations of the harmony degree in multifactor harmony and multilevel harmony will be discussed.

### 4.1. Harmony Degree Equation of a Factor

The harmony degree of a given factor is defined by the following equation:

(2) $$\begin{array}{c} {\text{HD}}_{p}=ai-bj, \end{array}$$

where HD_*p*_ is the harmony degree corresponding to a certain factor and HD_*p*_ ∈ [0,1]. A higher value of HD_*p*_ (closer to 1) indicates a higher harmony degree. If the result of (2) shows that HD_*p*_ < 0, then HD_*p*_ is set to 0.

The variables *a* and *b* are the unity degree and the difference degree, respectively. The unity degree *a* expresses the proportion of harmony participants in accordance with harmony rules with the same goal. The difference degree *b* is the expression of the proportion of harmony participants with divergent harmony rules and goals. Note that *a* ∈ [0,1], *b* ∈ [0,1], and *a* + *b* ≤ 1. In the presence of “neither unity nor differences” (i.e., “waiver” phenomenon), *a* + *b* < 1; otherwise, *a* + *b* = 1. If the harmony actions of a given factor in *n*-participant harmony are “*A*_1_, *A*_2_,…, *A*_*n*_,” it is assumed that the harmony actions of the *n*-participant harmony with the same target are “*G*_1_, *G*_2_,…, *G*_*n*_”; thus, *a* = ∑_*k*=1_^*n*^ *G*_*k*_/∑_*k*=1_^*n*^ *A*_*k*_. If there is no waiver, then *b* = 1 − *a*. For example, if the harmony rule is *A*_1_ : *A*_2_ = 2 : 1 and *A*_1_ and *A*_2_ are 100 and 40, respectively, then *G*_1_ and *G*_2_ equal 80 and 40, respectively, *a* = (80 + 40)/(100 + 40) = 0.8571, and *b* = 1 − *a* = 0.1429. If *A*_1_ and *A*_2_ are 100 and 80, respectively, then *G*_1_ and *G*_2_ equal 100 and 50, respectively, *a* = (100 + 50)/(100 + 80) = 0.8333, and *b* = 1 − *a* = 0.1667.

The variable *i*, which is the harmony coefficient, represents the satisfaction degree of the harmony goals and can be determined based on the calculation of the harmony goals, *i* ∈ [0,1]. If the harmony goals are absolutely achieved, then *i* = 1. In contrast, if the goals are not achieved, then *i* = 0. The harmony coefficient curve or function can be determined based on the satisfaction degree.

The variable *j*, which is the disharmony coefficient that reflects the divergent harmony participants, can be calculated and determined according to the difference degree. Note that *j* ∈ [0,1]. If the harmony participants are completely opposed, then *j* = 1. In contrast, if the harmony participants are not opposed, then *j* = 0. In all other cases, the value of *j* is within the range of 0 to 1. The disharmony coefficient curve or function can be determined based on the difference degree; that is, the disharmony coefficient depends on the extent of opposition.

In single-factor harmony (i.e., *m* = 1), the harmony degree equation is expressed as the following equation:

(3) $$\begin{array}{c} \text{HD}=ai-bj. \end{array}$$

### 4.2. Harmony Degree Equation for Multifactor Harmony

If there are a number of factors in a harmony problem, a comprehensive multifactor harmony degree should be calculated based on the single-factor harmony degree. This can be accomplished through two methods: weighted average calculation and exponential weighted calculation.

#### 4.2.1. Weighted Average Calculation

Consider the following:

(4) $$\begin{array}{c} \text{HD}=\overset{m}{\underset{p=1}{\sum }}w_{p}{\text{HD}}_{p}, \end{array}$$

where HD is the comprehensive harmony degree, HD ∈ [0,1], *w*_*p*_ is the weight of each harmony degree, *w*_*p*_ ∈ [0,1], and ∑_*p*=1_^*m*^ *w*_*p*_ = 1. The other variables have the same definition as above.

#### 4.2.2. Exponential Weighted Calculation

Consider the following:

(5) $$\begin{array}{c} \text{HD}=\overset{m}{\underset{p=1}{\prod }}{\left({\text{HD}}_{p}\right)}^{\beta_{p}}, \end{array}$$

where *β*_*p*_ is the index weight of each harmony degree, *β*_*p*_ ∈ [0,1], and ∑_*p*=1_^*m*^ *β*_*p*_ = 1. The other variables have the same definition as before.

### 4.3. Calculation of Multilevel Harmony Degree

**Figure 1:**
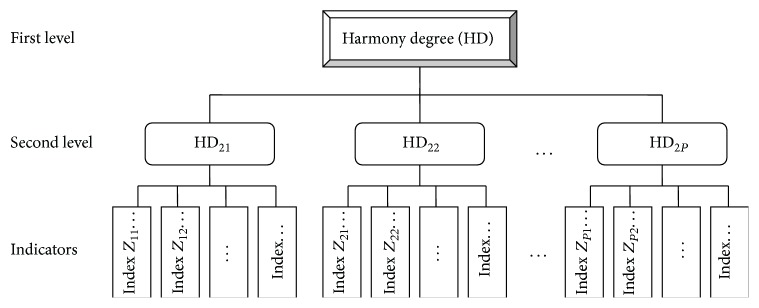
Multilevel harmony index system and harmony degree calculation.

There are complex multilevel harmony problems in real life, and a higher-level harmony problem (i.e., a more comprehensive harmony problem) includes or implies a set of lower-level harmony problems (i.e., single harmony problems). Therefore, the calculation of the harmony degree of harmony problems with different levels is essential.  Figure 1 shows a harmony problem with two levels. The first level is the highest and the harmony degree is HD, and the second level is a lower level that includes several harmony problems, which are expressed as HD_21_, HD_22_, …, HD_2*P*_ (*P* is the number of second-level harmony problems). Each lower-level harmony problem has corresponding indexes; that is, the indicators of HD_21_, HD_22_, and HD_2*P*_ are *Z*_11_, *Z*_12_,…, *Z*_21_, *Z*_22_,…, and *Z*_*P*1_, *Z*_*P*2_,…, respectively.

The calculation process of a multilevel harmony problem is as follows. (1) Calculate the harmony degree of the lowest-level harmony problem using the multifactor harmony degree method presented above. (2) Based on the results of step (1), calculate the harmony degree of a higher-level harmony problem in accordance with the weighted average or the exponential weighted method. For instance, as shown in Figure 1, HD = ∑_*p*=1_^*m*^ *w*_*p*_HD_2*p*_ or HD = ∏_*p*=1_^*m*^(HD_2*p*_)^*β*_*p*_^. (3) Repeat step (2) until the harmony degrees of the highest-level harmony problem are calculated.

## 5. Assessment of Harmony

The harmony assessment in water resources management represents the assessment of the harmony degree. This analysis can reflect the overall harmony degree, the present state and level of the harmony degree, and the space-time variation in the harmony degree. Thus, this assessment can provide insight into the evaluation of harmony problems and the development of a harmony strategy. The two main methods for harmony assessment are discussed.

### 5.1. Evaluation of the Harmony Degree

The evaluation of the harmony degree is a method in which the harmony degree is directly calculated according to certain problems to determine the level of the harmony degree based on its magnitude and to evaluate the calculated harmony degree.

### 5.2. Multi-Index Comprehensive Evaluation

Multi-index comprehensive evaluation is a method used to characterize the harmony degree synthetically through the establishment of a set of evaluation indexes and criteria. It includes the following three steps: (1) to establish an index system; (2) to determine the evaluation criteria; and (3) to select the evaluation and calculation methods. There are various types of multi-index comprehensive evaluation methods, such as the fuzzy comprehensive evaluation method, the gray comprehensive evaluation method, the analytic hierarchy process method, the set pair analysis method, and the matter element analysis method.

## 6. Harmony Regulation

Harmony regulation, which is primarily based on the harmony assessment, involves the use of some measures to improve the harmony degree. The primary task of harmony regulation is to advance the harmony degree to ultimately move the harmony problem in a more harmonious direction.

There are two thoughts in harmony regulation. The simple thought is a direct selection in accordance with the magnitude of the harmony degree, that is, “optimal selection method of the harmony action set.” The complex thought is to obtain the optimal harmony scheme through the development of harmony regulation models, namely, “optimization-based models of the function of the harmony degree.”

### 6.1. Optimal Selection Method of the Harmony Action Set

The optimal selection method of a harmony action set is to gather all of the harmony actions that meet a certain target (i.e., form a harmony action set) and then select the needed harmony actions (or schemes) from the set (i.e., obtain a concentrated optimal set of harmony actions).

If the harmony degree of the selected harmony action is the maximum centralized harmony degree, then the selected harmony action is considered the optimal harmony action. If it is difficult to obtain the maximum harmony degree, a suboptimal action, which is called a quasi-optimal harmony action, can be used.

Therefore, the key steps of this method are as follows: (1) combine many different schemes (or harmony actions) and calculate the harmony degree for each scheme using the abovementioned harmony degree calculation methods and (2) combine all of the harmony action sets that coincide with the relevant target values and select the optimal harmony action or the approximately optimal harmony action intensively.

This method has two effects on harmony regulation: (1) the optimization of harmony actions and (2) the optimization of harmony regulation. Through the harmony degree calculation of multiple schemes, access to the maximum or near-maximum harmony degree is easy, which contributes to the optimal scheme selection. However, it would also be easy to select the most favorable harmony regulation by changing a variety of possible options. In fact, sometimes the best harmony rule has a significant effect on the harmony problem.

### 6.2. Optimization-Based Model of the Function of the Harmony Degree

The development of an optimization model is a common calculation method used in operational research and systems science and has been used widely in practice. A general optimization model consists of an objective function and a set of constraints, and the general form of an optimization model is expressed as follows:

(6) Z=max⁡⁡FX,  GX≤0, X≥0,

where *X* is a decision vector, *F*(*X*) is the objective function, the variable *Z* is the maximum value of the objective function (note that the minimum can be transformed into the maximum by taking the negative of both sides), and *G*(*X*) is a set of constraints, which should be written such that the value of each specific constraint is less than or equal to 0 in the equation (if the constraint condition is greater than or equal to 0, it can be transformed to less than or equal to 0 by taking the negative).

This method can be used for the following three conditions.

(1) Establish an optimization model using the harmony degree equation as the objective function. This model is primarily used to identify the optimal harmony action (optimization scheme) under the condition that the harmony degree is the maximum possible value. The normal method for using the harmony degree equation as the objective function is
  (7) Z=max⁡⁡HDX,  GX≤0, X≥0.
(2) Construct an optimization model based on the harmony degree as a constraint. This model is primarily used to identify an optimization scheme that ensures that the harmony degree is above a certain limit. This method requires that the harmony degree be not less than a given limit value (set as *u*_0_) and has the following form:
  (8) Z=max⁡⁡FX,  GX≤0,  HD(X)⩾u0,KKKKkkkkkkkkkkkkkkkkkkkkkkkkkkKKX≥0.
(3) Optimize the harmony regulation. Set up an optimization model that uses the relevant parameters as a variable; that is, set the harmony regulation variable as *Y*. The general form of the optimization problem is then the following:
  (9) Z=max⁡⁡FX,Y,  GX,Y≤0, X,Y≥0.

## 7. Application Examples

### 7.1. Harmony Theory Description of the “Tragedy of the Commons”

The “tragedy of the commons,” which is a famous example of game theory, cannot be explained well by game theory alone. However, it can be commendably solved using harmony theory.

It is assumed that there is a field of grass that is shared by two families (*A* and *B*) for the raising of sheep. Families *A* and *B* have 6 and 3 members, respectively. In addition, family *A* has *n*_*A*_ sheep, and family *B* has *n*_*B*_ sheep. There is no doubt that a certain amount of grass is essential for all of the sheep to survive, and the total number of sheep has an upper limit.

The relevant assumptions are as follows. The harmony goal of this problem is to ensure that the grassland is controlled such that its grazing capacity is not destroyed. If the normal growth of grass exhibits the general requirement of *n*_*A*_ + *n*_*B*_ ≤ 300, then all of the grass would be destroyed if the number of sheep reaches 400. The harmony regulation is that the number of raised sheep is proportional to the population; that is, *n*_*A*_ : *n*_*B*_ = 2 : 1. Under this condition, it is optimal that families *A* and *B* raise 200 and 100 sheep, respectively. However, what is the harmony situation in other cases? Various assumptions are analyzed below.

**Figure 2:**
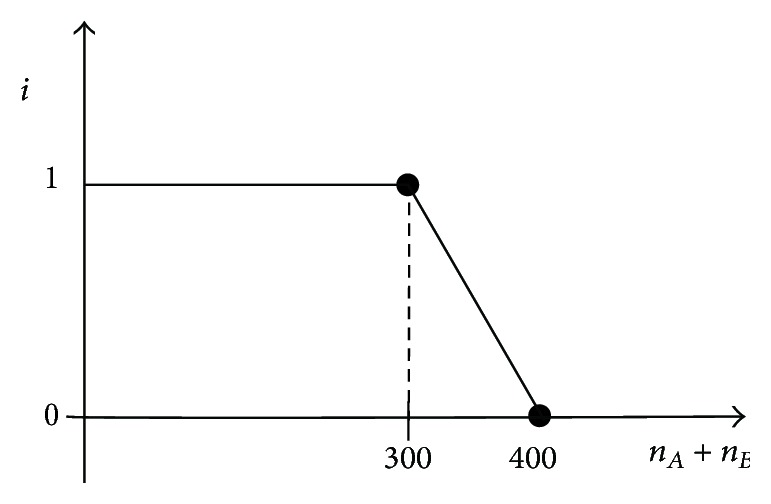
Function of the harmony coefficient *i*.

**Figure 3:**
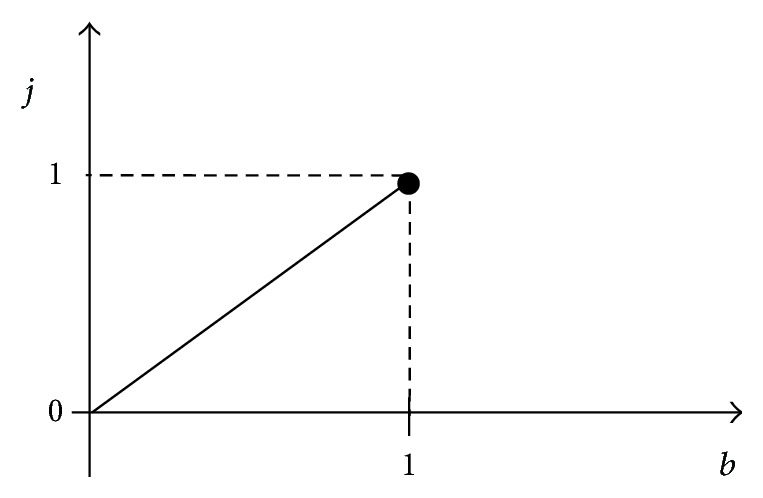
Function of the disharmony coefficient *j*.

(1) First, list the function of the harmony coefficient *i* according to the harmony goals, as shown in Figure 2. Second, determine the function of the disharmony coefficient *j*, as shown in Figure 3.

According to the harmony regulation that the number of raised sheep is proportional to the population (i.e., *n*_*A*_ : *n*_*B*_ = 2 : 1), the results are as follows. If *A* owns 200 sheep and *B* owns 100 sheep, the harmony action of *A* and *B* with the same goal is 200 and 100, respectively. Then, *a* = (200 + 100)/(200 + 100) = 1 and *b* = 0. In contrast, if *A* has 200 sheep and *B* has 160 sheep, the harmony action of *A* and *B* with the same goal is still 200 and 100. Then, *a* = (200 + 100)/(200 + 160) = 0.83 and *b* = 1 − *a* = 0.17. If *A* raises 200 sheep and *B* raises 60 sheep, the harmony action of *A* and *B* with the same goal is 120 and 60. Then, *a* = (120 + 60)/(200 + 60) = 0.69 and *b* = 1 − *a* = 0.31.

(2) Compare *n*_*A*_ + *n*_*B*_ with the harmony objectives and calculate the harmony coefficient *i* according to the function of the harmony coefficient *i*. Similarly, calculate the disharmony coefficient *j* in accordance with divergence *b* and its function.

(3) Calculate the harmony degree for several scenarios, as shown in Table 1. The final conclusion is as follows: the optimal harmony action is *n*_*A*_ : *n*_*B*_ = 2 : 1 and *n*_*A*_ + *n*_*B*_ ≤ 300.

The following assumptions were made. If there are no requirements on the harmony regulation, a harmony action is optimal as long as *n*_*A*_ + *n*_*B*_ ≤ 300 and the harmony degree is 1, which indicates that there are no requirements on the divergence between the harmony participants. If the harmony regulation is *n*_*A*_ : *n*_*B*_ = 2 : 1, a harmony action is optimal only when *n*_*A*_ = 2 × *n*_*B*_ and *n*_*A*_ + *n*_*B*_ ≤ 300. As shown in Table 1, scenarios 1, 5, and 6 are the optimal harmony actions according to the definition, and scenario 1 is certainly the best scheme with the maximum benefit.

### 7.2. Optimization of Water Allocation

Transboundary water distribution (regional water allocation) is a very important issue in hydraulic engineering practice. Due to the limit and scarcity of water resources, conflicts appear frequently between regions. As a result, the reasonable distribution of water has long been a difficult issue discussed by the academic community.

Assume that the known study area is divided into three partitions (A, B, and C) and the amount of available water is 764 million cubic meters. In addition, the water diversion proportion is assumed to be 4 : 4 : 2, and the population of the three partitions is 1.49, 1.34, and 0.75 million, respectively, which results in a total population of 3.58 million. Moreover, the average total outputs per cubic meter of water attained by the three partitions are 96, 112, and 105 yuan, respectively.

It is assumed that two harmony factors need to be considered. One is the water distribution harmony factor, which takes the requirements of water resources distribution into account according to the harmony regulation of the proportion of water distribution. The other harmony factor is the benefit harmony factor, which takes the benefit requirements brought by the water resources into consideration in accordance with the harmony regulation of equality in the per capita output.

#### 7.2.1. Function of the Harmony Degree and Harmony Assessment

For the unity degree calculation under the first harmony factor (i.e., water distribution), calculate the unity degree *a* based on harmony actions *G*_1_, *G*_2_, and *G*_3_ that meet the harmony regulation. As a result, *a* = ∑_*k*=1_^*n*^ *G*_*k*_/∑_*k*=1_^*n*^ *A*_*k*_, where *n* = 3.

To calculate the unity degree for the second harmony factor (i.e., harmony benefit factor), it is assumed that the per capita output of the three partitions is equal; thus, the unity degree *a* is 1. If these were not equal (*x*_1_, *x*_2_, and *x*_3_ are assumed separately), the unity degree *a* can be calculated according to the exponential weighted calculation of equal weight with the ratio of each value to the maximum using the following formula:

(10) $$\begin{array}{c} a=\sqrt[{3}]{\frac{x_{1}\times x_{2}\times x_{3}}{{\left[\max\left(x_{1},x_{2},x_{3}\right)\right]}^{3}}}. \end{array}$$

To satisfy the first harmony factor, the total amount of distributed water must be less than the available water resources; that is, the harmony coefficient *i* equals 1 when this objective is met, and *i* = 0 if this objective is not met. Furthermore, if the influence of the disharmony coefficient is not considered, *j* = 0.

There are no specific harmony objectives for the benefit harmony factor. The harmony coefficient *i* equals 1, and the disharmony coefficient *j* is 0.

The multifactor harmony degree (Formula (5)) is calculated taking the two harmony factors into account and using the exponential weighted calculation of equal weights. The results including the final multifactor harmony degree of 38 schemes with different water distributions throughout the three partitions (i.e., harmony actions of this issue) are listed in Table 2.

#### 7.2.2. Optimization of the Harmony Action

In this section, the optimization problem seeks to identify the most optimal harmony action that results in the highest harmony degree. The 38 schemes in Table 2 essentially reflect the process of seeking an optimal harmony behavior. The overall process is the following. First, calculate the multifactor harmony degree (Scheme 1) in accordance with the agreed-upon proportion for the water distribution. Second, judge the direction of the water distribution amount for the three partitions that makes the multifactor harmony degree increase and determine an approximate range for the optimal solution (calculated according to a step of 0.1). For example, it is obvious that the harmony degree of Scheme 15 is the maximum from Schemes 2 to 19; in this scheme, the amounts of water allocated to the three partitions are 340, 270, and 154 million m^3^. Third, obtain the optimal harmony action, which is represented with scenario 26, in which the amounts of water allocated to the three partitions are 333, 270, and 161 million m^3^, respectively, based on changes to the water allocation distribution in scenario 15 (the step size was decreased to 0.01). The harmony degree of scenario 26 is 0.9319, which indicates that this scenario can be described as “basic harmony,” that is, approximately complete harmony.

#### 7.2.3. Optimization of the Harmony Rule

In this section, the optimization problem seeks to identify the optimal harmony rule (i.e., water distribution harmony rule) based on changes in the proportion used for the water distribution. In this example, changing the water distribution proportion implies changing the water rules and the calculation methods. The procedures used to calculate the harmony degree are unchanged. The fundamental difference between this and the previous optimization is the changing harmony rules. The harmony regulation used in the previous calculation is a water distribution proportion of 4 : 4 : 2, whereas this proportion is changed in the following analysis.

Table 3 shows the optimal harmony action and harmony degree calculated with changing harmony regulations (i.e., water distribution proportion). The corresponding optimal harmony action and harmony degree can be obtained using similar steps (Table 2); the only difference is that the harmony regulation (water distribution proportion) is changed repeatedly. For example, the harmony rule in Scheme 1 (Table 3) is 3.36 : 2.68 : 1.6, and the corresponding amounts of water allocated to the three partitions are 335, 269, and 160 million cubic meters, respectively. The water distribution proportion was calculated with a step size of 0.01, and some of the calculation results are listed in Table 3. Scheme 4 exhibits the maximum harmony degree of 0.9889 with the optimal harmony rule of 3.35 : 2.69 : 1.60. This maximum harmony degree is significantly larger than that obtained in the previous analysis (Table 2), which demonstrates that the overall level can be improved through the optimization of the harmony regulations.

## 8. Conclusions

This paper illustrates the widespread existence of harmony relationships and demonstrates that a quantitative study of harmony issues is of great significance for the analysis of various relationships in nature and human society. This is achieved through the introduction of the five essential factors of harmony theory, the calculation of the harmony degree and a harmony assessment, the discussion of harmony regulation issues, and the solution of two application examples.

Through the expositions and the two application examples, some conclusions can be obtained. (1) Harmony issues are common phenomena in nature and human society, and the use of quantitative research is of vital importance. (2) The harmony degree equation is a quantitative expression of harmony issues and a basic mathematical equation used to calculate the dimensions of the harmony degree. A harmony degree HD of 1 indicates complete harmony, whereas HD = 0 indicates absolute disharmony. A value of HD between 0 and 1 indicates changes in the harmony degree from absolute disharmony to complete harmony in accordance with the quantitative expressions of the harmony degree. (3) The optimal harmony action, the optimal harmony rule, and the best management solution can be obtained mathematically, which provides a theoretical basis for the solutions of many practical problems. (4) As an emerging subdiscipline, harmony theory will aid the scientific understanding and arrangement of harmony issues. Further research on the quantitative expressions and assessment of the harmony degree and the search for optimal harmony regulation strategies will provide additional insight into harmony issues.

## Figures and Tables

**Table 1:** Harmony degree calculation for various scenarios of the “tragedy of the commons.”

Scenario	*n*_*A*_	*n*_*B*_	*a *	*b *	*I *	*j *	HD	Directions
1	200	100	1	0	1	0	1	*n*_*A*_ : *n*_*B*_ = 2 : 1 and *n*_*A*_ + *n*_*B*_ ≤ 300. Optimal action.
2	120	100	0.82	0.18	1	0.18	0.79	Certain “differences” occur.
3	200	80	0.86	0.14	1	0.14	0.84	Certain “differences” occur.
4	200	60	0.69	0.31	1	0.31	0.59	Larger “differences” occur.
5	160	80	1	0	1	0	1	*n*_*A*_ : *n*_*B*_ = 2 : 1 and *n*_*A*_ + *n*_*B*_ ≤ 300. Optimal action.
6	100	50	1	0	1	0	1	*n*_*A*_ : *n*_*B*_ = 2 : 1 and *n*_*A*_ + *n*_*B*_ ≤ 300. Optimal action.
7	200	150	0.86	0.14	0.5	0.14	0.41	Harmony goal is exceeded, and some “differences” occur.
8	250	100	0.86	0.14	0.5	0.14	0.41	Harmony goal is exceeded, and some “differences” occur.
9	300	150	1	0	0	0	0	Harmony goal is significantly exceeded.
10	250	180	0.87	0.13	0	0.13	0	Harmony goal is significantly exceeded, and some “differences” occur.

**Table 2:** Harmony degree calculation for different schemes of water allocation.

Scheme	Water of partition A (billion m^3^)	Water of partition B (billion m^3^)	Water of partition C (billion m^3^)	Harmony degree of the water diversion harmony factor	Harmony degree of the benefit harmony factor	Multifactor harmony degree
1	3.06	3.06	1.52	0.9948	0.8624	0.9262
2	3.50	2.50	1.64	0.8181	0.9633	0.8877
3	3.40	2.50	1.74	0.8181	0.9171	0.8662
4	3.30	2.50	1.84	0.8181	0.8748	0.8460
5	3.20	2.50	1.94	0.8181	0.8359	0.8269
6	3.10	2.50	2.04	0.8181	0.7998	0.8089
7	3.00	2.50	2.14	0.8181	0.7663	0.7918
8	3.50	2.60	1.54	0.8508	0.9731	0.9099
9	3.40	2.60	1.64	0.8508	0.9666	0.9068
10	3.30	2.60	1.74	0.8508	0.9200	0.8847
11	3.20	2.60	1.84	0.8508	0.8773	0.8639
12	3.10	2.60	1.94	0.8508	0.8380	0.8443
13	3.00	2.60	2.04	0.8508	0.8015	0.8258
14	3.50	2.70	1.44	0.8835	0.9629	0.9223
15	3.40	2.70	1.54	0.8835	0.9752	0.9282
16	3.30	2.70	1.64	0.8835	0.9691	0.9253
17	3.20	2.70	1.74	0.8835	0.9221	0.9026
18	3.10	2.70	1.84	0.8835	0.8790	0.8813
19	3.00	2.70	1.94	0.8835	0.8393	0.8611
20	3.39	2.70	1.55	0.8835	0.9763	0.9288
21	3.38	2.70	1.56	0.8835	0.9775	0.9293
22	3.37	2.70	1.57	0.8835	0.9786	0.9298
23	3.36	2.70	1.58	0.8835	0.9797	0.9304
24	3.35	2.70	1.59	0.8835	0.9808	0.9309
25	3.34	2.70	1.60	0.8835	0.9818	0.9314
**26**	**3.33**	**2.70**	**1.61**	**0.8835**	**0.9829 **	**0.9319 **
27	3.32	2.70	1.62	0.8835	0.9790	0.9301
28	3.31	2.70	1.63	0.8835	0.9741	0.9277
29	3.35	2.69	1.60	0.8802	0.9853	0.9313
30	3.34	2.69	1.61	0.8802	0.9839	0.9306
31	3.33	2.69	1.62	0.8802	0.9788	0.9282
32	3.32	2.69	1.63	0.8802	0.9738	0.9259
33	3.31	2.69	1.64	0.8802	0.9689	0.9235
34	3.35	2.71	1.58	0.8868	0.9763	0.9305
35	3.34	2.71	1.59	0.8868	0.9774	0.9310
36	3.33	2.71	1.60	0.8868	0.9784	0.9315
37	3.32	2.71	1.61	0.8868	0.9795	0.9320
38	3.31	2.71	1.62	0.8868	0.9793	0.9319

**Table 3:** Optimal harmony action and harmony degree for different schemes of water allocation with varied harmony rules (proportion of water distribution).

Scheme	Water distribution proportion			Optimal harmony actions			Multifactor harmony degree
	(harmony rules)			(amount of water allocated, billion m^3^)			
	Partition A	Partition B	Partition C	Partition A	Partition B	Partition C	
1	3.36	2.68	1.60	3.35	2.69	1.60	0.9874
2	3.36	2.69	1.60	3.35	2.69	1.60	0.9881
3	3.35	2.68	1.60	3.35	2.69	1.60	0.9883
**4**	**3.35**	**2.69**	**1.60**	**3.35**	**2.69**	**1.60**	**0.9889**
5	3.34	2.69	1.61	3.34	2.69	1.61	0.9879
6	3.33	2.69	1.62	3.34	2.69	1.61	0.9848
7	3.32	2.69	1.63	3.34	2.69	1.61	0.9818
8	3.31	2.69	1.64	3.34	2.69	1.61	0.9788
9	3.30	2.69	1.65	3.34	2.69	1.61	0.9758
10	3.36	2.70	1.58	3.36	2.69	1.59	0.9871
11	3.35	2.70	1.59	3.35	2.69	1.60	0.9871
12	3.34	2.70	1.60	3.35	2.69	1.60	0.9871
13	3.33	2.70	1.61	3.33	2.70	1.61	0.9871
14	3.34	2.71	1.59	3.33	2.70	1.61	0.9853
15	3.33	2.71	1.60	3.33	2.70	1.61	0.9853
